# The Characterization of Novel Tissue Microbiota Using an Optimized 16S Metagenomic Sequencing Pipeline

**DOI:** 10.1371/journal.pone.0142334

**Published:** 2015-11-06

**Authors:** Jérôme Lluch, Florence Servant, Sandrine Païssé, Carine Valle, Sophie Valière, Claire Kuchly, Gaëlle Vilchez, Cécile Donnadieu, Michael Courtney, Rémy Burcelin, Jacques Amar, Olivier Bouchez, Benjamin Lelouvier

**Affiliations:** 1 Vaiomer SAS, Labège, France; 2 INRA, GeT-PlaGe, Genotoul, Castanet-Tolosan, France; 3 INRA, UAR1209, Castanet-Tolosan, France; 4 INRA, UMR1388, GenPhySE, Castanet-Tolosan, France; 5 INSERM U1048, I2MC, Toulouse, France; 6 Rangueil Hospital, Department of Therapeutics, Toulouse, France; Charité, Campus Benjamin Franklin, GERMANY

## Abstract

**Background:**

Substantial progress in high-throughput metagenomic sequencing methodologies has enabled the characterisation of bacteria from various origins (for example gut and skin). However, the recently-discovered bacterial microbiota present within animal internal tissues has remained unexplored due to technical difficulties associated with these challenging samples.

**Results:**

We have optimized a specific 16S rDNA-targeted metagenomics sequencing (16S metabarcoding) pipeline based on the Illumina MiSeq technology for the analysis of bacterial DNA in human and animal tissues. This was successfully achieved in various mouse tissues despite the high abundance of eukaryotic DNA and PCR inhibitors in these samples. We extensively tested this pipeline on mock communities, negative controls, positive controls and tissues and demonstrated the presence of novel tissue specific bacterial DNA profiles in a variety of organs (including brain, muscle, adipose tissue, liver and heart).

**Conclusion:**

The high throughput and excellent reproducibility of the method ensured exhaustive and precise coverage of the 16S rDNA bacterial variants present in mouse tissues. This optimized 16S metagenomic sequencing pipeline will allow the scientific community to catalogue the bacterial DNA profiles of different tissues and will provide a database to analyse host/bacterial interactions in relation to homeostasis and disease.

## Introduction

Animal cells coexist with a complex ecosystem of bacteria and archaea. This microbiota, which outnumbers eukaryotic cells at least tenfold [1], is mostly present in the gastrointestinal tract and at other epithelial surfaces such as the skin, oral cavity, lung mucosa and vagina [1–3]. A large body of evidence demonstrates the importance of epithelial bacteria in the maintenance of health [4,5]. Recent studies are consistent with the existence of microbiota in diverse tissues and organs such as the liver, adipose tissue, blood and atheroma plaque and these bacteria may play a role in non-infectious pathologies [6–10]. Importantly, the function of this microbiota could impact the physiology of the tissue. For example, gram-negative bacteria in adipose tissue from obese patients [11] are responsible for the triggering of pre-adipocyte precursors and macrophage proliferation [12].

Identifying the bacterial taxa (living bacteria or bacterial DNA) present within tissues will aid in elucidating the molecular mechanisms implicated in the control of cellular and physiological functions of the host. The exhaustive study of tissue microbiota requires culture-independent methods such as metagenomic sequencing. 16S rDNA-targeted metagenomic sequencing (also referred to as 16S metagenomics or 16S metabarcoding) allows the analysis of the relative proportion of bacterial taxa in a sample using specific amplification by PCR of the 16S ribosomal RNA gene (16S) coupled to next generation high throughput sequencing (NGS). Whereas for a number of years Roche 454 pyrosequencing has been the gold standard for 16S metagenomics [13,14], the release of the MiSeq kit reagents v2 (2x250 bp pair ended reads) and v3 (2 x300 bp pair ended reads) by Illumina, permitted for the first time the use of the MiSeq technology to reach an amplicon length compatible with 16S metagenomics. MiSeq technology combines several major advantages compared to 454 technology: i) higher output (8.5 Gb for kit v2 and 15 Gb for kit v3) allowing more exhaustive analysis of complex microbiota and/or more samples per sequencing run ii) lower cost per read and iii) a simplified procedure for library construction. Technical limitations exist that hamper the metagenomic analysis of tissue microbiota, including high abundance of PCR inhibitors and other eukaryotic products, which complicate dramatically the extraction and sequencing of bacterial DNA present within the samples [15,16]. This study describes the design, and validation of an optimized 16S metagenomics pipeline to investigate taxonomic diversity in tissue microbiota using MiSeq reagent kits v2 and v3 and presents its application in the analysis of microbiota in liver, muscle, heart, brain and adipose tissue. In addition to protocol optimization for tissue sample, we designed the pipeline with several specificities to reduce cost and complexity, and to facilitate the adaptation of the method to new primers and future technical improvements from Illumina.

Deciphering the tissue microbiota will help to identify the molecular crosstalk between the host and the bacteria and will thus lay the groundwork for the understanding of homeostatic and pathological mechanisms and the identification of novel therapeutic strategies.

## Materials and Methods

### Sample preparation and DNA extraction

#### BEI mock communities

Genomic DNA from microbial mock communities B, HM-782D (v5.1L, even, low concentration) and HM-783D (v5.2L staggered, low concentration) were obtained from BEI Resources (NIAID, NIH as part of the Human Microbiome Project, Manassas, VA, USA). HM-783D contains genomic DNA mixture from 20 bacterial strains containing staggered ribosomal RNA operon counts (1,000 to 1,000,000 copies per organism per μl). HM-782D contains genomic DNA from the same 20 bacterial strains with equimolar (even) ribosomal RNA operon counts (100,000 copies per organism per μl). See S1 Table for bacterial strain list.

#### Designed mock community

The designed mock community was prepared by cloning the complete 16S rDNA gene of 14 different bacterial species. Genomic DNA from *Acinetobacter johnsonii* (NCIMB 8154), *Aminobacter aminovorans* (NCIMB 9039), *Devosia riboflavin* (NCIMB 8177), *Eubacterium barkeri* (NCIMB 10623), *Geofilum rubicundum* (NCIMB 14482), *Paracoccus denitrificans* (NCIMB 8944), *Prosthecobacter fusiformis* (NCIMB 12777) and *Xanthomonas sp*. (NCIMB 592) were obtained from NCIMB (Aberdeen, Scotland). Genomic DNA from *Lactococcus lactis* (CIRMBP-611) was obtained from CIRM-BP (INRA UMR 1282 ISP, Nouzilly, France). Bacterial strains *Bifidobacterium animalis subsp*. *lactis*, *Cupriavidus necator (Ralstonia eutropha)*, *Escherichia coli*, *Ralstonia mannitolilytica*, *and Ralstonia pickettii* were provided by Dr Remy Burcelin (Inserm/UPS UMR 1048—I2MC, Toulouse France). The genomic DNA of these 5 bacterial strains was extracted using the Trizol method following the protocol recommended by the manufacturer (Life Technologies, Grand Island, NY, USA). The complete 16S rRNA gene of the 14 bacterial strains was amplified using the 8F (AGAGTTTGATCCTGGCTCAG) [17] and 1489R (TACCTTGTTACGACTTCA) [18] primers. The PCR products were purified using the NucleoSpin^®^ Gel and PCR Clean-up (Macherey-Nagel, Düren, Germany) and cloned into *Escherichia coli* TOP10F using the pCR2.1 TOPO TA cloning kit (Life Technologies). Recombinant clones were verified by checking the insert size by PCR with the M13F (GTAAAACGACGGCCAG) and M13R (CAGGAAACAGCTATGAC) primers, by restriction fragment length polymorphism with NotI and SpeI restriction enzymes (New England Biolabs, Ipswich, MA, USA), and by Sanger sequencing (GATC Biotech, Constance, Germany) of both strands (1480 bp, using the 16S rDNA gene of *E*. *coli* as a reference, although the length varies depending on the organisms [19,20]). The validated recombinant clones were then cultured in liquid LB medium supplemented with ampicillin (65 μg/ml) overnight at 37°C. After centrifugation (5 min 8000 g at 4°C), plasmids were extracted using the PureLink® Quick Plasmid Miniprep Kit (Life Technologies). Following the plasmid purification, a second verification of the inserted DNA sequence (including PCR, RFLP and Sanger sequencing as described above) was performed for final plasmid validation. For the designed mock community mixture, the concentration of each plasmid extract was defined using NanoDrop 2000 UV spectrophotometer (Thermo Scientific, Waltham, MA, USA). See Table 1 for the complete bacterial strain list. The genomic DNA of the strains of *Bifidobacterium animalis subsp*. *lactis*, *Cupriavidus necator* (*Ralstonia eutropha*), *Escherichia coli*, *Ralstonia mannitolilytica*, and *Ralstonia pickettii*, extracted for the designed mock community mixture preparation, were also used to prepare several mixes of genomic bacterial DNA, using NanoDrop 2000 UV spectrophotometer to set the proportions of each strains in the mix.

**Table 1:** Designed mock community.

Species	Family	Phylum	Copies of16S rDNA
*Acinetobacter johnsonii *	Moraxellaceae	Proteobacteria (Gamma)	10^7^
*Aminobacter aminovorans*	Phyllobacteriaceae	Proteobacteria (alpha)	10^7^
*Bifidobacterium animalis subsp*. *lactis *	Bifidobacteriaceae	Actinobacteria	10^7^
*Cupriavidus necator* (= *Ralstonia eutropha*)	Burkholderiaceae	Proteobacteria (Beta)	10^7^
*Devosia riboflavina*	Hyphomicrobiaceae	Proteobacteria (alpha)	10^7^
*Escherichia coli*	Enterobacteriaceae	Proteobacteria (Gamma)	10^7^
*Eubacterium barkeri*	Eubacteriaceae	Firmicutes	10^7^
*Geofilum rubicundum*	Marinilabiliaceae	Bacteroidetes/Chlorobi	10^7^
*Lactococcus lactis*	Streptococcaceae	Firmicutes	10^7^
*Paracoccus denitrificans*	Rhodobacteraceae	Proteobacteria (alpha)	10^7^
*Prosthecobacter fusiformis*	Verrucomicrobiaceae	Chlamydiae/Verrucomicrobia	10^7^
*Ralstonia mannitolilytica*	Burkholderiaceae	Proteobacteria (Beta)	10^7^
*Ralstonia pickettii*	Burkholderiaceae	Proteobacteria (Beta)	10^7^
*Xanthomonas sp*.	Xanthomonadaceae	Proteobacteria (Gamma)	10^7^

#### Tissue samples

In addition to fecal samples, we choose six tissue samples representing diverse organs: ileum, liver, skeletal muscle, heart, brain and mesenteric adipose tissue (MAT). Tissue samples were collected from C57-BL/6J mice (Charles River, Wilmington, MA, USA) raised in a specific pathogen-free animal facility. Samples were directly frozen in liquid nitrogen and stored at -80°C until DNA extraction.

For DNA extraction, to access most of the bacterial DNA present in the tissues (free DNA or in living /dormant/degraded bacteria, circulating or inside eukaryotic cells) without damaging the DNA, we tested empirically numerous protocols of lysis (mechanical and/or enzymatic). The lysis protocol that gave the best yield of bacterial DNA extraction from fecal and tissue samples consisted of a mechanical disruption step for 5 seconds using Turax (KA, Germany) followed by a lysis step using acid-washed glass beads (Sigma, Saint-Louis, MO, USA) and Tissue Lyser (Qiagen, Venlo, Netherlands) for 2x 3 min at 30 Hz. After this lysis step, the total genomic DNA was extracted using the QIAamp DNA Stool kit (Qiagen) for fecal samples and Trizol (Life Technologies) for all other tissues according to the manufacturer’s instructions. The quality and quantity of DNA extracts were analyzed by agarose gel electrophoresis (1% agarose in TBE 0.5X) and NanoDrop 2000 UV spectrophotometer (Thermo Scientific).

### Primer design and library preparation

**Fig 1:**
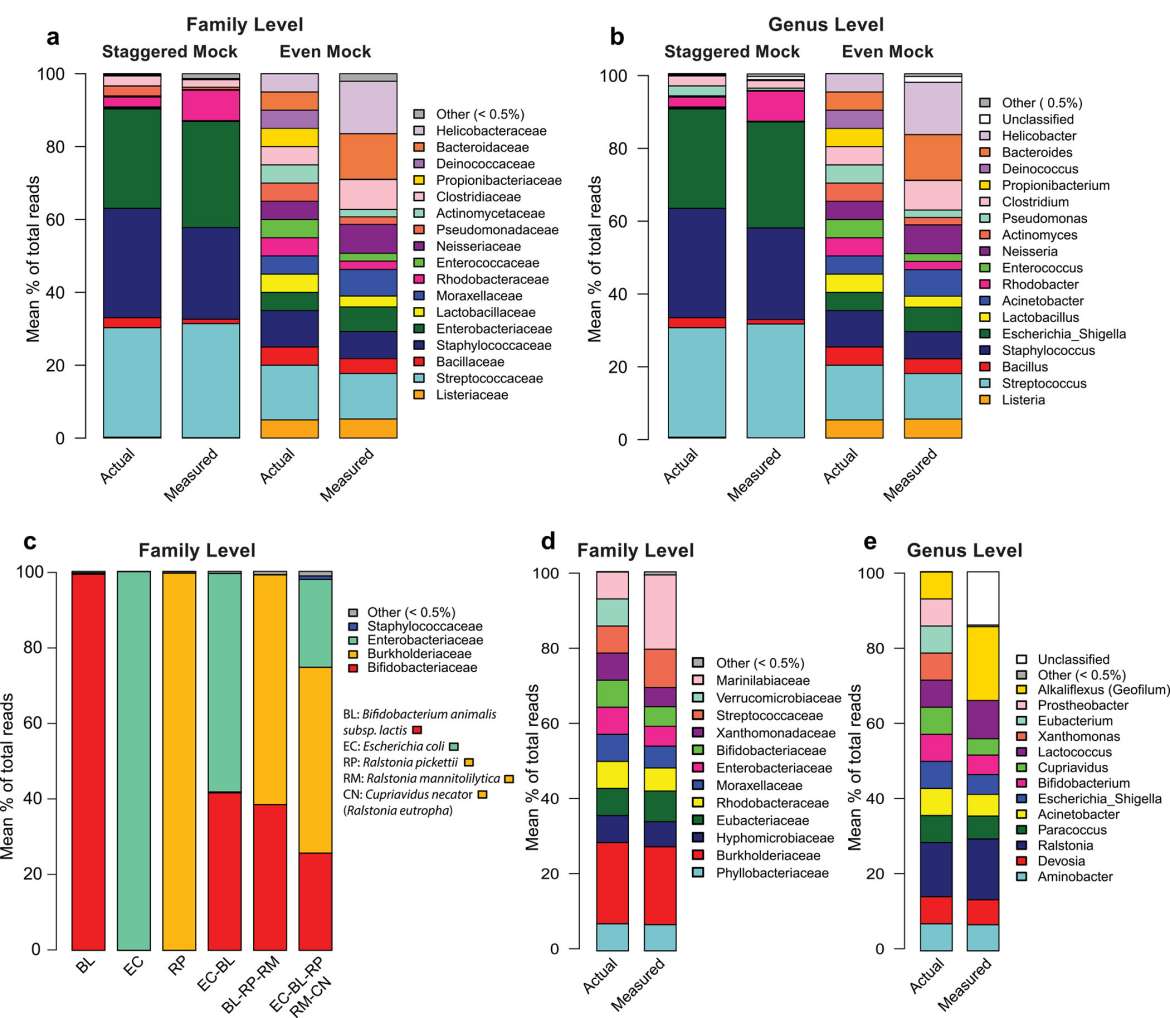
Pipeline validation against mock communities. **(a)** Stacked bar charts showing the actual relative abundance of bacterial families in the staggered and even BEI genomic mock communities and the measured relative abundance of the families obtained with the MiSeq sequencing pipeline. **(b)** The relative abundance as in **a**, but at the genus taxonomic level. **(c)** Relative abundance of the families obtained by sequencing genomic DNA of a single strain of bacteria or simple mix of bacterial genomic DNA. **(d)** Stacked bar charts showing the actual relative abundance of bacterial families in the plasmid based mock community and the measured relative abundance of the families obtained with the MiSeq sequencing pipeline. **(e)** The relative abundance as in **d**, but at the genus taxonomic level. The sequencing was performed in triplicate for all the samples (starting from the extracted DNA); the means of the triplicates are shown on the stacked bar charts.

**Fig 2:**
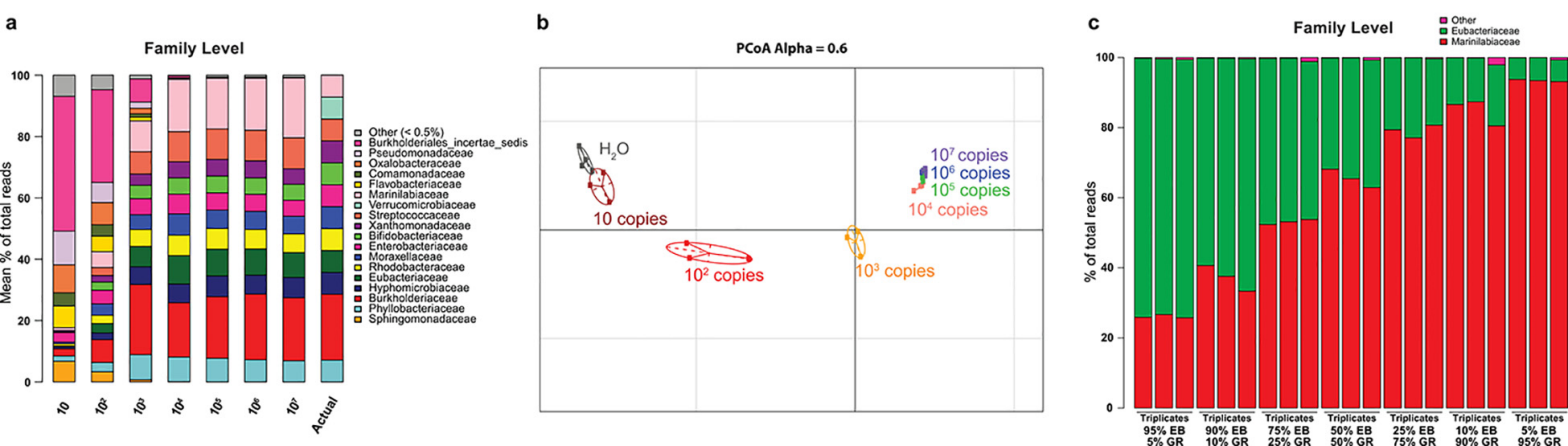
Pipeline sensitivity and assessment of the relative abundance accuracy. **(a)** Stacked bar charts showing the relative abundance of bacterial families obtained by sequencing of a serial dilution of the plasmid-based mock community (from 10 and 10^7^ copies of 16S gene for each of the 14 bacterial strains) compared to the actual relative abundance (right stacked bar). **(b)** Generalized UniFrac distance based PCoA analysis of the sequencing of a serial dilution of the plasmid-based mock community shown in a compared with the negative control generated by sequencing molecular biology-grade water with the same pipeline (H_2_O). UniFrac weight parameter (Alpha) was set to 0.6 for this analysis. **(c)** Stacked bar charts showing the relative abundance of bacterial families obtained by sequencing 7 mixes with different ratios of two of the bacterial plasmids containing the 16S gene of *Geofilum rubicundum* (GR) and *Eubacterium barkeri* (EB). The sequencing was performed in triplicate for all the samples (starting from the extracted DNA); the means of the triplicates are shown in **a** and individual triplicates are shown in **b** and **c**.

**Fig 3:**
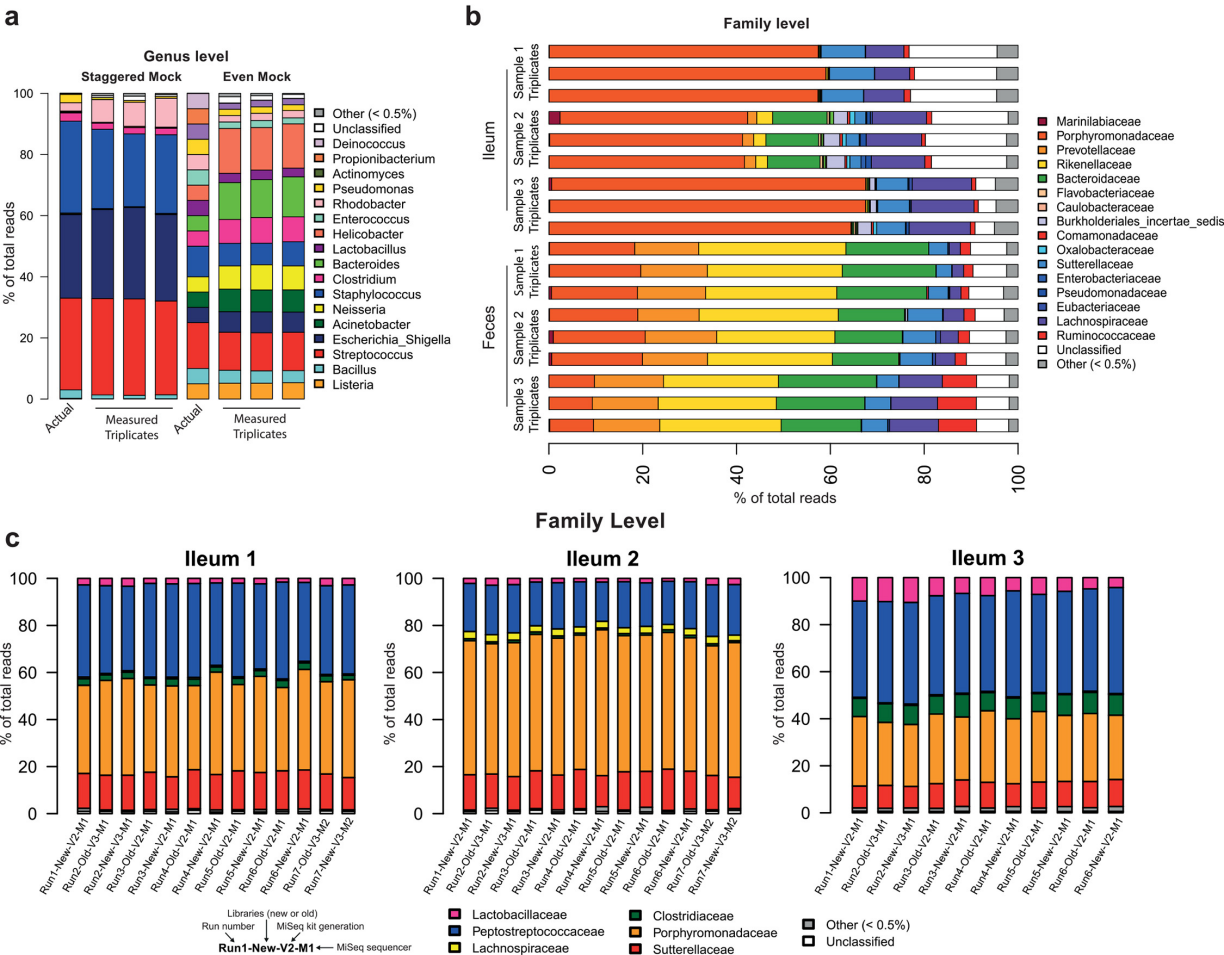
Replicability and reproducibility. **(a)** Stacked bar charts showing the actual relative abundance of bacterial families in the staggered and even BEI genomic mock communities and the measured relative abundance in triplicates of the families obtained with the MiSeq sequencing pipeline. **(b)** Stacked bar charts showing the relative abundance of bacterial families obtained by sequencing of triplicates of fecal samples and ileum mucosa samples collected from three different mice (Sample 1, 2 and 3). **(c)** Stacked bar charts showing the relative abundance of bacterial families obtained by sequencing of three samples of mouse ileum mucosa in six to seven runs each with different parameters described in the legend at the bottom left: different runs, new libraries from same extracted DNA or the same libraries already prepared, different MiSeq kit generation and different sequencers. Two different experimenters performed the different runs and reagent batch numbers (including Taq polymerase) varied from run to run.

**Fig 4:**
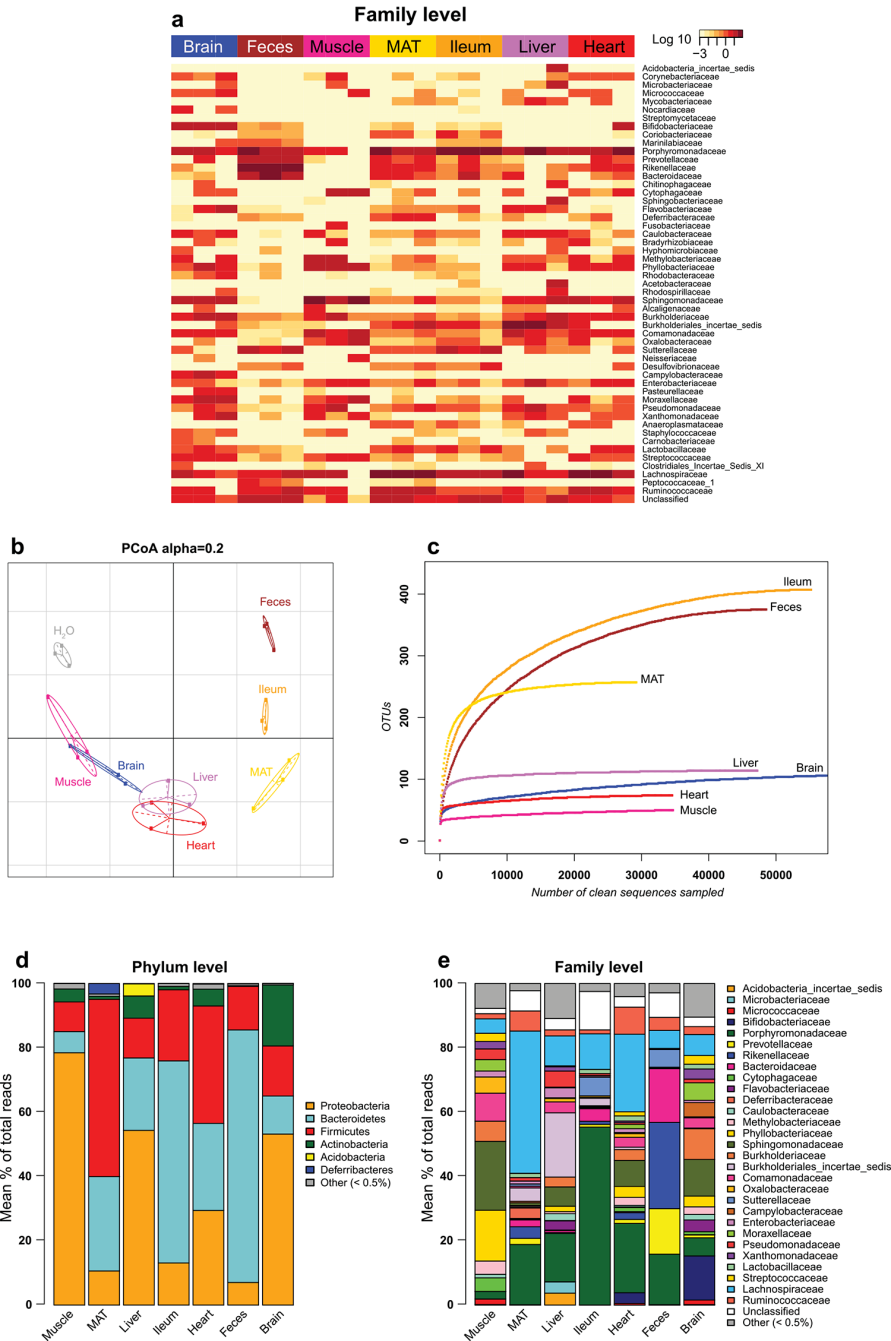
16S metagenomics on diverse tissue samples. **(a)** Heatmap of the relative abundance of each bacterial family from sequencing of different mouse tissue samples performed in triplicate (three different mice for each tissue). Each line corresponds to a bacterial family; each one of the three columns for a tissue corresponds to a different mouse. **(b)** Generalized UniFrac distance-based PCoA analysis of sequencing data from the samples shown in **a** compared with the negative control generated by sequencing molecular biology-grade water with the same pipeline (H_2_O). UniFrac weight parameter (Alpha) was set to 0.2 for this analysis. **(c)** Rarefaction curve of the sequencing of the samples shown in **a** and **b**. For each tissue, only the sample with the median number of OTU is displayed. (**d)** Stacked bar charts showing the relative abundance of bacterial phyla obtained by sequencing of the mouse samples shown in **a**, **b** and **c**. **(e)** The relative abundance as in **d**, but at the family taxonomic level. MAT: Mesenteric adipose tissue. OTU: Operational Taxonomic Unit.

Except mentioned otherwise, all samples (Figs 1–4, S1 and S2 Figs) were analyzed in triplicate starting from the extracted DNA. The replicates presented were technical except in Fig 3B which displays both technical and biological replicates (3 mice with three technical replicates each) and Fig 4 which only presents biological replicates (three mice for each tissue). Negative controls to assess technical background were performed using Nuclease-free water (Ambion, LifeTechnologies) either in place of the tissue sample during the extraction step (lysis + trizol protocol), or in place of the extracted DNA during the library preparation. Each triplicate underwent all library preparation steps, sequencing and bioinformatics analysis, as described below. The V3-V4 hyper-variable regions of the 16S rDNA gene were amplified from the DNA extracts during the first PCR step using universal primer Vaiomer 1F (CTTTCCCTACACGACGCTCTTCCGATCT-TCCTACGGGAGGCAGCAGT, partial P5 adapter–primer) and universal primer Vaiomer 1R (GGAGTTCAGACGTGTGCTCTTCCGATCT-GGACTACCAGGGTATCTAATCCTGTT, partial P7 adapter–primer) which are fusion primers based on the qPCR primers designed by Nadkarni et al. [21]. Primers Vaiomer 1F and 1R include specificity for the 16S rDNA gene of 95% of the bacteria in the Ribosomal Database Project and part of the P5/P7 adapter targeted by the second PCR step (CTTTCCCTACACGAC and GGAGTTCAGACGTGT). Our primer design and 2 step PCR strategy allow shorter primers which are more suitable for the amplification of bacterial DNA extracted from tissue samples carrying large amounts of PCR inhibitors and eukaryotic DNA. This PCR was performed using 2 U of a DNA-free Taq DNA Polymerase and 1x Taq DNA polymerase buffer (MTP Taq DNA Polymerase, Sigma). The buffer was complemented with 10 nmol of dNTP mixture (Euromedex, Souffelweyersheim, France), 15 nmol of each primer (Sigma) and Nuclease-free water (Ambion, Life Technologies) in a final volume of 50 μl. The PCR reaction was carried out on a Veriti Thermal Cycler (Life Technologies) as follows: an initial denaturation step (94°C for 10 min), 35 cycles of amplification (94°C for 1 min, 68°C for 1 min and 72°C for 1 min) and a final elongation step at 72°C for 10 min. Amplicons were then purified using the magnetic beads Agencourt AMPure XP—PCR Purification (Beckman Coulter, Brea, CA, USA) following the 96 well format procedure modified as follow: beads/PCR reactional volume ratio of 0.8 x and final elution volume of 32 μl using Elution Buffer EB (Qiagen). The concentration of the purified amplicons was controlled using Nanodrop 8000 spectrophotometry (Thermo Scientific).

Sample multiplexing was performed using tailor-made 6 bp unique indexes, which were added during the second PCR step at the same time as the second part of the P5/P7 adapters used for the sequencing step on the MiSeq flow cells with the forward primer Vaiomer 2F (AATGATACGGCGACCACCGAGATCTACACT-CTTTCCCTACACGAC, partial P5 adapter–primer targeting primer 1F) and reverse primer Vaiomer 2R (CAAGCAGAAGACGGCATACGAGAT-index-GTGACT-GGAGTTCAGACGTGT, partial P7 adapter including index–primer targeting primer 1R). This second PCR step was performed on 50–200 ng of purified amplicons from the first PCR using 2.5 U of a DNA free Taq DNA Polymerase and 1x Taq DNA polymerase buffer. The buffer was complemented with 10 nmol of dNTP mixture (Euromedex), 25 nmol of each primer (Eurogentec, HPLC grade) and Nuclease-free water (Ambion, Life Technologies) up to a final volume of 50 μl. The PCR reaction was carried out on a Veriti Thermal Cycler (Life Technologies) and ran as follow: an initial denaturation step (94°C for 10 min), 12 cycles of amplification (94°C for 1 min, 65°C for 1 min and 72°C for 1 min) and a final elongation step at 72°C for 10 min. Amplicons were purified as described for the first PCR round. The concentration of the purified amplicons was measured using Nanodrop 8000 spectrophotometry (Thermo Scientific) and the quality of a set of amplicons (12 samples per sequencing run) was tested using Agilent DNA 7500 chips using the Bioanalyzer 2100 (Agilent Technologies, Santa Clara, CA, USA). Controls were carried out to ensure that the high number of PCR cycles (35 cycles for PCR 1 + 12 cycles for PCR2) did not create significant amounts of PCR chimera or other artifacts. The region of the 16S rDNA gene to be sequenced has a length of 467 bp for a total amplicon length of 522 bp after PCR 1 and of 588 bp after PCR 2 (using the 16S rDNA gene of *E*. *coli* as a reference). All libraries were pooled in the same quantity in order to generate equivalent number of raw reads with each library. The DNA concentration in the pool (no dilution, diluted 10x and 20x in EB + Tween 0.5% buffer) was quantified by qPCR using the 7900HT Fast Real-Time PCR System (Life Technologies) and KAPA Library Quantification Kits for Illumina Platform (Kapa Biosystems, Inc., Wilmington, MA, USA) as recommended by the manufacturer (Illumina, San Diego, CA, USA). The final pool, at a concentration after dilution between 5 and 20 nM, was used for sequencing.

### Sequencing

The pool was denatured (NaOH 0.1N) and diluted to 7 pM. The PhiX Control v3 (Illumina) was added to the pool at 15% of the final concentration as described in the Illumina procedure. 600 μl of this pool and PhiX mixture were loaded onto the Illumina MiSeq cartridge according to the manufacturer’s instructions using MiSeq Reagent Kit v2 (2x250 bp Paired-End Reads, 8.5 Gb output) or MiSeq Reagent Kit v3 (2x300 bp Paired-End Reads, 15 Gb output, only for samples marked as V3 in Fig 3C and S2E Fig. FastQ files were generated at the end of the run to perform the quality control. The quality of the run was checked internally using PhiX Control and then each pair-end sequence was assigned to its sample using the multiplexing index.

### Bioinformatics analysis

#### New MiSeq Indexes

In order to increase the number of samples multiplexed on the same MiSeq run, new indexes were designed using two steps implemented in Perl scripts. The first step had the following rules: i) index length must be 6 bases in the alphabet ['A', 'C', 'G', 'T'], ii) each index pair must exhibit at least 2 mismatches and iii) the sequence of each index pair must not be reverse complement nor complement. The first step resulted in 466 new indexes. To avoid any mismatch and allow a proper calibration of the MiSeq camera, we selected before each run in the second step up to 320 optimal indexes (S2 Table) having i) minimum similarities between them, ii) equivalent proportions of A/C and T/G (corresponding to the two excitation LED of the MiSeq sequencer) and iii) proportions of each base at each position close to 25% among all the indexes. To reduce even more the risk of miss-assignment of reads to samples, we did not allow during demultiplexing any mismatch in the index sequences.

#### Sequencing Data Preprocessing

The raw MiSeq sequencing data were processed using the NG6 application [22] for demultiplexing without mismatches on the index sequence and FASTQ generation using Illumina CASAVA v1.8.2 on the GenoToul computer cluster (68 nodes, each node consists of 40 core/80 threads CPU and 256 GB of RAM). The determination of a quality threshold to trim the reads based on the Illumina quality scores is difficult due to overestimation of the error probability for low quality base calls [23]. Therefore, we did not apply any *a priori* filters based on the Illumina quality scores to avoid unneeded loss of good sequence bases. The quality filters implemented in this approach are mainly based on the pair joining quality and the random nature of sequencing errors. Moreover, the quality trimming based on Illumina scores can reduce the length of the reads to the extent that overlap is lost, rendering them unusable for the rest of the analysis. To overcome this difficulty and to filter out poor quality reads, read pair joining was performed using FLASH (v1.2.7) with default values except for the following constraints i) reduce base mismatches between the 2 reads in the overlapping region by setting the maximum mismatch density to 0.1 ii) limit the minimum length of the overlap to 10 for 2x250bp reads and to 110 for 2x300 bp reads iii) set the maximum overlap length to 70 for 2x250bp reads and to 170 for 2x300 bp reads. The resulting FASTQ files for successfully joined pairs were converted to FASTA format using FASTX Toolkit (v0.0.14) and merged into a single file. This file constitutes our sequence working set.

#### 16S Metagenomic Analysis Pipeline

The bioinformatics pipeline used for the 16S metagenomics studies in tissues was based on the protocol published by Kozich et al. [24] and has similarities with the protocol developed in parallel by Unno [25], with adjustments for specific difficulties presented by the analysis of tissue microbiota and to adapt to the short overlap of our paired-end reads. The pipeline, run on an Intel Xeon server (16 cores/32 Threads CPU and 208 GB of RAM), is composed of the following steps:

*Step 1*: *Sequence Filtering and Trimming*. The previous step produces sequences of different lengths, which is not suited for sequence clustering into Operational Taxonomy Units (OTU). Non-specific PCR amplification could also have occurred especially with tissue samples containing high concentrations of both eukaryotic and non-eukaryotic DNA. In order to address these issues, the Mothur analysis environment v1.33.0 [26] was used first to de-replicate (i.e. to cluster redundant sequences) the sequence working set obtained in the previous step and align the non-redundant sequences to the SILVA bacteria reference 16S alignment (v102) distributed with Mothur. The sequences that do not align to the reference 16S alignment are considered as non-specific PCR amplification and therefore culled from the sequence working set. The remaining aligned sequences were trimmed to obtain blunt ends to allow sequence clustering.

*Step 2*: *Sequencing Error Reduction*. As the OTU clustering could be dramatically affected by sequencing errors, and although the Illumina MiSeq technology has a low error rate, quality filtering is required to obtain accurate amplicon sequences. Contrary to the Roche 454 technology, the insertion-deletion errors with MiSeq are negligible (<0.001%). Substitution errors are more frequent (0.1%) and have to be tackled. The substitutions occur at random positions and at extremely low frequencies, making them relatively easy to detect when sequence coverage is sufficiently deep. We took advantage of this feature to aggregate rare sequences with more abundant and closely related groups of sequences. The Mothur pre.cluster command achieves this by merging low frequency sequences with very close higher frequency sequences using a modified single linkage algorithm. Three differences were allowed during this clustering step. At the end of this pre-clustering step, the singletons (unclustered sequences) were considered as either uncaught sequencing errors or extremely rare taxa with abundances under the background level of the method and were withdrawn from the sequence working set.

*Step 3*: *PCR Chimera Removal*. The chimeras were screened *de-novo* as described in the MiSeq SOP (August 2013, http://www.mothur.org/wiki/MiSeq_SOP), i.e. considering the most abundant sequences to build a reference of non-chimeric sequences, using UCHIME v4.2 [27] and removed from the sequence working set.

*Step 4*: *Taxonomic Assignment of Sequences*. The Mothur implementation of the Naive Bayesian Classifier [28] was run against the RDP rRNA training set (v9) to provide a taxonomic assignment to every sequence with a minimum bootstrap confidence score of 80%. The sequences that were not assigned to the Bacteria domain were filtered out for the rest of the analysis (including mitochondrial and chloroplast DNA). The primers specificity ensured that no sequences corresponding to mitochondria or chloroplast were found during this filtering.

*Step 5*: *Clustering into OTU*. The clustering of sequences was performed using the average neighbor algorithm on our clean sequence working set as described in the MiSeq SOP at a threshold of 0.03% identity. Finally, a consensus taxonomy is provided for each OTU based on the taxonomic assignment of individual reads using the default cutoff (51%).

For the samples sequenced in this study, we obtained on average 146,987 raw sequences (raw read pairs) per sample. 119,413 sequences (81.24% of the raw sequences) were kept on average after the manufacturer passing filters, we obtain 88,868 sequences on average (74.4% of the manufacturer passing filters pairs) after pair joining and related quality filters and finally 60,979 sequences on average (51.1% of the manufacturer passing filters pairs) after OTU clustering and related quality filters.

### Statistics, data analysis and figures preparation

Custom R and Perl scripts were used to perform the data acquisition from Mothur output files, the statistical data analysis and generate the figures.

The 16S metagenomics profiles are displayed using heatmaps and barplots. Heatmaps were generated using the gplots CRAN library (v2.11.0) to plot the relative abundance of sequences for each sample assigned to the taxa at the different taxonomic levels. For better visualization of low abundant taxa, the data were log10-transformed. Barplots represent the proportion of sequences classified to taxa at the different taxonomic levels. The proportions presented are either those calculated on single samples or averages calculated over different sequencing triplicates. For clarity, the low abundant taxa (mean proportion <0.5%) have been grouped into a single category called “other (<0.5%)”. The 'unclassified' category represents the sequences that could not be assigned to a taxon at a given taxonomic level with at least 80% confidence score.

The dissimilarities between the 16S profiles of samples were analyzed using the principal coordinate analysis (PCoA). The Generalized UniFrac distance [29] was calculated using the GUniFrac CRAN library (v1.0) to generate the distance matrix. The alpha parameter, controlling the weight assigned to the most abundant taxa, is specified on each figure. For 16S rich samples such as stool or intestinal tract samples, the potential 16S contaminants contributed by the PCR mix is negligible. However, for more challenging samples such as tissue samples, negative target controls (molecular biology grade water sequenced with the same metagenomic pipeline) were added to the PCoA to ensure that the potential amplification of contaminants do not impact the sequencing data of our samples.

To determine the suitable sequencing depth for each tissue, a rarefaction curve was plotted for each sample. The rarefaction curve plots the number of OTU as a function of the number of sequences sampled in the clean sequence working set. We used a step of 100 sequences for the X axis and sampled each step 1000 times to calculate the average number of OTU plotted on the Y axis. For better readability, only the median curve for each tissue is shown.

Sequencing data are available on the European Nucleotide Archive (ENA) under the study ID “PRJEB10949” at http://www.ebi.ac.uk/ena/data/view/PRJEB10949. Custom R and Perl scripts are available on sourceforge at http://sourceforge.net/projects/tissue-microbiota/files/

### Ethics Statement

Studies on mice were performed in accordance with the article R-214-89 of the French “Code rural et de la pêche maritime” section 6 “Use of living animal for scientific research” and approved by the ethical committee CEEA-122 of the SICOVAL Prologue Biotech Institute.

## Results

### Overall strategy and primer choice

The metagenomics pipeline comprises: 1) a DNA extraction step optimized to maximize the recovery of bacterial DNA from tissue samples; 2) a sequence library construction method based on a two-step PCR using short primers (the first PCR to target the 16S sequence, the second PCR to add a single index and the Illumina adapters; 3) the sequencing step on MiSeq and 4) a tailor-made bioinformatics processing of the data. We sequenced amplicons of 467 bp encompassing the V3 and V4 variable regions of the 16S gene since these variable regions are reported to have the broadest phylogenetic information for studying the microbiota of human and other mammalian species [30–32].

We used diverse mouse tissues and mock bacterial communities to test the pipeline and analyze the potential of the MiSeq technology for 16S metagenomics analysis of animal tissues. We performed numerous controls both *in vitro* and *in silico* to ensure the absence of artefacts such as cross-contamination between tissue samples or amplification of bacterial DNA contaminants from reagents. Indeed, many reagents required in the sequencing pipeline, including most of the TAQ polymerases available on the market, contain non negligible amounts of bacterial DNA [33–35]. A significant part of the pipeline optimization involved testing combinations of reagents to minimize bacterial contaminants and adapting the protocol to increase the yield of extraction and amplification of the bacterial DNA present in the tissue. The protocols of tissue dissection and DNA extraction were also carefully designed to minimize any risk of contamination between different tissue samples.

### Pipeline validation using BEI mock communities

First, we assessed the ability of the sequencing pipeline to detect bacterial taxa in the mock communities from BEI Resources (S1 Table) and to assign the corresponding OTU at the genus and family levels. We tested both the staggered (1,000 to 1,000,000 copies of RNA operon per organism per μl depending on the organisms) and even mock communities (100,000 copies of operon counts per organism per μl for each organism). All the bacteria strains present in the mock communities were sequenced and assigned to the family and genus levels (Fig 1A and 1B), except *Deinococcus radiodurans* strain R1: an unusual extremophile environmental bacteria of the phylum *Deinooccus-Thermus* [36] and for which the 16S gene cannot be amplified by the primer pair we designed (as predicted by *in silico* validation). *Propionibacterium acnes* was sequenced and assigned to the family and genus levels, but reads assigned to *Propionibacterium acnes* represented less than 0.5% of the total reads and therefore fell into the “Other” category on the barplots.

The PCR amplification, sequencing steps and bioinformatics processing create a biased distribution of the observed community in comparison to the actual distribution of the mock communities provided by BEI. This biased distribution depends on the 16S region amplified, the PCR primers and the proportion of each strain in the mock community [37–39]. The measured abundance of each strain at the genus level differs from the actual abundance by 0.4% to 9.7% (Median = 3.0) and by 0.02% to 6.7% (Median = 0.2) in the even and the staggered mock communities respectively (Fig 1A and 1B).

### Pipeline validation using designed mock communities

The mock communities from BEI are used as standards by many research groups for molecular microbiology and metagenomic sequencing. This type of mock community is a useful tool to compare performance with published studies. Most of the organisms of the BEI mock communities are particularly common bacterial strains that are well represented in the taxonomic databases used in bioinformatics. However, biological tissue samples contain mostly less common bacteria that are poorly represented or absent from the available databases. For this reason, we also tested the pipeline using mock communities composed of random bacteria representative of different phyla of interest (Table 1). We purposely included strains of bacteria absent from the RDP database (*Geofilum rubicundum*, a genus and species of bacteria discovered in 2012 [40]) and strains of Verrucomicrobia (*Prosthecobacter fusiformis*), a phylum that cannot be amplified with our primers according to *in silico* simulations. We sequenced several mixes of these bacterial DNAs, either genomic DNA (Fig 1C) or plasmids incorporating the 16S genes of different strains of bacteria (Fig 1D and 1E). Genomic DNA is more similar to tissue samples and the use of plasmid-based mock communities allows precise quantitation of the number of 16S gene copies included in the mix.

The separate sequencing of the genomes of *Bifidobacterium animalis subsp*. *lactis*, *Ralstonia pickettii* and *Escherichia coli* resulted in assignment to the corresponding bacterial families (Bifidobacteriaceae, Burkholderiaceae, and Enterobacteriaceae) for 99.3%, 99.5%, and 99.9% of the reads respectively (Fig 1C).

A 1:1 genomic DNA mix of *Escherichia coli* and *Bifidobacterium animalis subsp*. *lactis* resulted in an assignment of respectively 41.6% and 57.7% to the corresponding family (Enterobacteriaceae and Bifidobacteriaceae) (Fig 1C). The deviation from 50% is explained by i) a degree of unavoidable bias introduced by the sequencing pipeline and ii) the variation in 16S copy number per genome and genome size between bacteria [20,41].

A 1:1:1 genomic DNA mix of *Bifidobacterium animalis subsp*. *lactis*, *Ralstonia picketti* and *Ralstonia mannitolilytica* resulted in assignments of 37.0% to the *Bifidobacterium animalis subsp*. *lactis* family (Bifidobacteriaceae) and 59.5% to the family of the *Ralstonia* species (Burkholderiaceae) (Fig 1C).

Finally, we mixed and sequenced the same genomic DNA proportion (20%) of *Escherichia coli*, *Bifidobacterium animalis subsp*. *lactis* and three Burkholderiaceae: *Ralstonia picketti*, *Ralstonia mannitolilytica and Cupriavidus necator*. The resulting assignments were: 23.2% Enterobacteriaceae, 25.7% Bifidobacteriaceae and 49.0% Burkholderiaceae (Fig 1C).

We then tested a mix of 14 plasmids each of which carried a copy of the 16S gene of different strains (Table 1). Except for *Prosthecobacter fusiformis*, which, as confirmed by *in silico* analysis, cannot be amplified by the primers used, we identified all strains at the family level, including the recently discovered *Geofilum rubicundum* (Fig 1D), and 11 strains at the genus level (Fig 1E). The *Geofilum* genus is absent from the RDP database and is considered by the Naive Bayesian classifier as a bacterium from the *Alkaliflexus* genus, the genus closest to *Geofilum*[40]. The assigned proportions of the different bacteria were generally very close to the real copy numbers present in the samples (Fig 1D and 1E).

### Pipeline versatility: example of primer optimization

We took advantage of the versatility of the pipeline to design and incorporate a new set of primers to amplify the Verrucomicrobia phylum in addition to the bacterial taxa recognized by the former set of primers (≥ 95% of Ribosomal Database Project). With the new set of primers, all 14 strains of the designed mock community, including *Prosthecobacter fusiformis*, were sequenced and assigned to the correct family and 12 of them to the genus level (S1 Fig **)**.

### Pipeline sensitivity

We investigated the bias of the MiSeq pipeline by assessing the impact of sample dilution and variation of the relative proportions of bacterial 16S genes. We sequenced serial dilutions of the plasmid-based mock community to obtain between 10 and 10^7^ copies of 16S gene for each of the 14 bacterial strains (Fig 2A and 2B). Between 10^7^ copies and 10^4^ copies of the 16S gene, the relative proportions were consistent and close to the actual values as shown on the bar plot (Fig 2A) and PCoA analysis (Fig 2B). However, at 10^3^ copies or lower, the measured proportions progressively divert from the actual composition and the technical background (mainly arising from bacterial DNA contaminants present in the TAQ polymerase) represented an increasing fraction of the reads (Fig 2A) and became closer to the profile of the negative control (H_2_O) (Fig 2B).

#### Accuracy of assessment of relative abundance

We sequenced seven mixes with different ratios of bacterial plasmids containing the 16S genes of *Geofilum rubicundum* and *Eubacterium barkeri*. The observed ratios corresponded consistently to the actual ratio hierarchy (Fig 2C) with some variations depending on the strains and the ratio of the plasmids. With *Geofilum rubicundum*, low proportions were overestimated: 5% *Geofilum rubicundum* generated 26.1% of the assigned reads, whereas 95% of the same plasmid produced a much more accurate ratio (93.4%) (Fig 2C). The bar plots showed that triplicates of each mix were consistent for all ratios (between 0.3% and 7.3% of variability, median = 3.8) (Fig 2C).

### Replicability and reproducibility

We analyzed the replicability of the pipeline during the same sequencing run (Fig 3A and 3B and S2A–S2D Fig) and between runs (Fig 3C, S2E Fig). For assessing the variability within the same run, all samples were treated in triplicate (whole metagenomic pipeline starting from extracted DNA). As shown in Fig 3A and 3B, the variability of the pipeline between triplicates is very low (median difference = 0.01 read % ± SEM 0.01) for all sample types (mock communities or tissues samples) and is independent of the bacterial taxa considered, their relative abundance or the taxonomy level. We also tested the reproducibility between several MiSeq runs on the same samples of ileum mucosa. Three samples of mouse ileum mucosa were sequenced on six to seven runs each using either the same library preparations (named “old”) or by generating new libraries (named “new”) from the extracted DNA samples (Fig 3C). We performed the different sequencing runs over a period of three months using either MiSeq Kit V2 (2x250bp) or V3 (2x300bp) and a different set of indexes. Moreover, the last run was performed on a different MiSeq desktop sequencer from the six previous runs. As shown on the bar plots (Fig 3C, S2E Fig) and the PCoA analysis (S3 Fig), variability between runs was very low (median difference = 0.01% of read ± SEM 0.02).

The same ileum samples (Fig 3C, S2E Fig), with the same 467 bp amplicon size, sequenced either with the MiSeq Kit v2 (2x250 bp) or Miseq Kit V3 (2x300 bp) gave the same results, despite the difference of overlap between the read pairs (33 bp for MiSeq V2 and 133 bp for MiSeq V3, using the 16S rDNA gene of E. coli as a reference). These show that the small overlap with the MiSeq V2 kit did not affect the quality of the results.

### 16S metagenomics on diverse tissue samples

Finally, we tested the pipeline by sequencing mouse samples from different organs and tissues: feces, ileum, liver, skeletal muscle, heart, brain and mesenteric adipose tissue (MAT). Each tissue was isolated from three different mice. Heat map representation (Fig 4A) and PCoA analysis (Fig 4B) showed that each sample was successfully sequenced and displayed a 16S metagenomic profile distinct from the technical background: H_2_O added during the library preparation (Fig 2B, Fig 4B and S4 Fig **)** or at the beginning of the extraction step (S4 Fig). Thus, the tissue profiles observed were not the result of an artefact due to the amplification of the bacterial DNA contaminating the reagents. Each tissue has a distinct taxonomic profile (which excludes the presence of a significant contamination between tissues) with similarities between certain tissues (e.g. liver and heart) (Fig 4A and 4B **)**. The high output of MiSeq sequencing, 20–45 million raw reads per run and between 30, 000 and 100, 000 sequences per sample (after filtering, cleaning, and assignation), generated high levels of relevant information and consequently the rarefaction analyses (Fig 4C) displayed an exhaustive description of the diversity of all tested samples. Some tissues, (e.g. feces, Ileum and adipose tissue), displayed a very high diversity in terms of bacterial content which could not have been assessed correctly with other lower throughput techniques. Bar plot representations of the results (Fig 4D and 4E) confirmed the previous observations and showed that the pipeline allowed effective assignment for this type of sample: 99.3% - 100% of assigned reads at the phylum level (Fig 4D) and 88.0% - 98.3% at the family level (Fig 4E). Assignments were more variable at the genus level, between 34.4% and 88.8%, probably reflecting a higher number of unknown genera in the reference taxonomy database for those samples (data not shown).

## Discussion

By employing an optimized MiSeq-based pipeline for the analysis of bacterial 16S rDNA, we have characterized the diversity of bacterial taxa present in mouse tissues.

This study opens new avenues for the understanding of host to microbiota interactions in relation to homeostasis and disease. 16S metagenomic sequencing techniques, first used in samples extremely rich in bacterial content such as environmental samples or feces, need specific optimizations to study more challenging samples like animal tissues that have lower levels of bacterial sequences and contain molecules that interfere with the DNA amplification methods [15,16]. Other groups have explored the potential of MiSeq-based methods for analyzing environmental and gut samples [24,42–44], but to our knowledge these have not previously been used to study microbiota in animal tissues. We have adapted and optimized the procedures provided by Illumina by introducing numerous unique modifications that are critical in order to obtain the sensitivity and robustness required to sequence the bacterial DNA present in animal tissues.

This optimized MiSeq pipeline is based on two PCR steps and a novel indexing strategy. The two-step PCR protocol allowed the use of shorter primers to target the 16S genes and to add the MiSeq adapters necessary for the sequencing steps (approximately 50 bases instead of up to 90 bases with a one-step PCR protocol). The shorter primers allow more efficient amplification of the samples, since long primers complicate the PCR cycle (higher annealing temperatures and increase of secondary structure formation) especially for challenging tissue samples. This strategy also allows the possibility of changing the specificity of the primer pairs without the need to redesign, resynthesize and re-optimize the primer pairs of the second PCR step. Indeed, the method allowed rapid modification of the primers to add the capability of sequencing the Verrucomicrobia phylum. With the same strategy, new primers can be efficiently designed to increase the length of the amplicon to exploit the increased read length offered by the new MiSeq Kit V3 (2x300 bp), target other regions of the 16S gene, other bacterial genes, or genomes from other kingdoms such as fungi. The second PCR step, which adds the indexes and sequencing adapters to the amplicons, can also be modified independently of the first step. Indeed we have replaced the original 180 indexes by 320 new indexes to take advantage of the higher throughput of the MiSeq v3 kit, without any need to modify or optimize the pipeline.

We extensively validated the pipeline with mock communities and negative controls to ensure the validity of the obtained results. We observed moderate differences between the actual proportions of 16S genes in the mock communities and the measured proportions assessed by sequencing. Indeed, as already reported, bias in the library preparation (PCR and cleaning), the sequencing steps and the bioinformatics analysis frequently introduce an over- or under-estimation of the proportions of a bacterial taxon, which depends on the individual taxa and their relative proportions in the samples [37–39,45]. However, the overall proportions were well respected, triplicates were consistent even between dilutions and progression was clearly observed when the proportion of a bacterial taxon was increased in the sample.

Because of the high diversity of the human microbiota and its variation among individuals, technologies with higher throughput than Roche 454 pyrosequencing are necessary to correlate microbiome composition with clinical parameters or disease states. The MiSeq technology, as assessed by the rarefaction analysis, allowed an exhaustive description of even the rich microbial population present in samples like feces and intestinal mucosa.

Another advantage of the MiSeq pipeline described here over 454-based sequencing protocols was the much higher reproducibility and replicability. The poor reproducibility of 454 technology between replicates and between runs has been noted in the literature [46–48]. Our MiSeq-based pipeline displayed a high reproducibility between replicates both within the same run and across runs. This reproducibility was manifest over broad time intervals and included sample triplicates from separate library preparations. This offers the important advantage of allowing robust comparisons of results between runs, for example for the analysis of large cohorts of samples in clinical trials or population studies.

In terms of read length and quality of assignment, studies have shown that despite higher read length, 454-based analyses do not exhibit a better depth of assignment than MiSeq [13,49]. Some studies point out that the type of mutations occurring with MiSeq (mainly substitutions), combined with a suitable quality filtering step allowed by read pairing makes this technology more reliable in terms of taxonomic assignment [24,50,51]. In our studies, MiSeq reached the required criteria in terms of quality and read depth with a taxonomic assignment at both the family and genus levels. We did not attempt to assign the reads and OTU to the species level, since NGS technologies that have an output sequence length of only several hundred base pairs cannot be used to assign with confidence the reads and OTU to species level except in a handful of specific cases [14,52–54].

Tests of sensitivity demonstrated the reliability and reproducibility of the pipeline down to 10^3^ copies of 16S rDNA in our samples. This corresponds to around 100–500 bacterial genomes in the sample, since each bacterium has about 1–15 copies of the 16S gene per genome [20,41]. In our experience, this level of sensitivity is sufficient for the analysis of human and animal tissue samples. With samples of low bacterial content, it is necessary to include negative controls in order to take into account the technical background during the analysis. All our negative controls (as illustrated in the different figures) demonstrate that the bacterial contaminants do not affect the quality of our results. In addition, the fact that each tissue sample has a specific and reproducible bacterial taxa profile supports the fact that the observed profile cannot be attributed to reagent contaminants or contaminants from other samples.

Because of technical difficulties and the belief that tissues and organs of healthy individuals do not contain bacteria, few studies have been undertaken to examine tissue bacteria except from epithelial surfaces [1,2,6–9]. We describe here the successful sequencing of microbiota from a variety of internal organs and identified reproducibly a bacterial DNA profile specific for each tissue. These findings raise important questions concerning the role of tissue microbiota in animal physiology. The presence of bacterial DNA in tissues does not necessarily imply the presence of living bacteria. Free bacterial DNA could be present in the intercellular space or most likely within cells, in particular immune cells. On the contrary, the reported absence of cultivable bacteria in healthy tissue is not synonymous with the absence of living bacteria in these tissues. Indeed, several technical limitations as well as biological features of living or dormant bacteria explain why the vast majority of microbial species from both environmental or tissue samples remain uncultivated [10,55,56]. Consequently, we cannot ascertain today what proportion of the bacterial DNA sequenced in tissues corresponds to living bacteria.

In addition to the tissue-specificity of the bacterial DNA profiles, a striking observation in our study was the alpha diversity richness in mesenteric adipose tissue. This supports the conclusions of several published studies concerning the involvement of host microbiota in obesity and diabetes [8,11,12,57–59]. The presence of a specific bacterial DNA profile in the brain is much more surprising since brain is assumed to be a sterile organ in the absence of disease. However, this observation, which was not possible before the development of next generation sequencing, is in accordance with previous studies that analyzed brain tissue from immunodepressive humans and rodents [60]. It would be interesting to investigate the potential role of bacteria or bacterial DNA present in the central nervous system in the control of the brain immune system by host microbiota [61,62] and in the development of neurodegenerative diseases [63–65].

In terms of bacterial DNA profile, we found in the gut (fecal and ileum samples) DNA belonging mostly to the Bacteroidetes and Firmicutes phyla, as previously shown by other groups in both mice and Humans [66–69]. Mesenteric adipose tissue also contains DNA mostly from Bacteroidetes and Firmicutes but not in the same proportion (more Firmicutes than Bacteroidetes). In addition, the mesenteric adipose tissue is the only tissue sample in our study shown to contain significant amounts of DNA belonging to the Deferribacters phylum.

Muscle, liver, heart and brain samples contain more DNA from the Proteobacteria and Actinobacteria phyla than the gut or adipose tissue. Liver is the only tissue to have DNA belonging to the Acidobacteria phylum. Overall, the bacterial DNA profiles of these non-intestinal tissues differ dramatically from those of the fecal or ileum samples, despite the fact that their microbiome derives at least partially (and probably mostly) from the gut as a result of bacterial translocation [70–73]. The difference in bacterial profiles between the gut and other tissues could be explained by the role of filter played by the intestinal and immune cells. This mechanism limits the translocation of a specific portion of the gut microbiota to the periphery. In addition, each tissue has its own specific bacterial DNA profile, but which is shared at least partially among individuals. The tissue specificity of the bacterial DNA profile could be explained both in terms of microbiology (living/dormant bacteria in a specific tissue environment, which is already demonstrated for example for bacterial species in blood [74–79]) and in terms of immunology (eukaryotic cells which could carry specific bacterial DNA depending on their location [80,81]). These mechanisms, as well as the physiological role of the bacteria and bacterial DNA present in the tissue, remain to be studied in detail.

## Conclusions

This enhanced method of 16S tissue metagenomic sequencing has permitted the first characterisation of bacterial DNA profiles in several animal tissues and will allow the scientific community to address the nature and role of tissue microbiota in human physiology and disease. These observations introduce the possibility of designing new approaches for the discovery of novel biomarkers and therapeutic targets.

## Supporting Information

S1 Fig Validation of the new primers against mock communities. **(a)** Stacked bar charts showing the actual relative abundance of bacterial families in the plasmid based mock community and the measured relative abundance of the families obtained with the MiSeq sequencing pipeline using either the original primers (described in the methods), or the new primers (designed to amplify also the Verrucomicrobia phylum). **(b)** The relative abundance as in **a**, but at the genus taxonomic level. The sequencing was performed in triplicate for all the samples (starting from the extracted DNA); the means of the triplicates are shown on the stacked bar charts.
(PDF)

S2 Fig Replicability and reproducibility. **(a-d)** Stacked bar charts showing in triplicates the actual relative abundance and the measured relative abundance of the families **(a, c)** and genus **(b, d)** of the BEI mock communities **(a, b)** and our own designed mock communities **(c, d)**. **(e)** Stacked bar charts showing the relative abundance of bacterial families obtained by sequencing of three samples of mouse ileum mucosa in six to seven runs each with different parameters described in the legend at the bottom left: different runs, new libraries from same extracted DNA or the same libraries already prepared, different MiSeq kit generations and different sequencers. Two experimenters performed the different runs and reagent batch numbers (including Taq polymerase) varied from run to run.
(PDF)

S3 Fig PCoA analysis of the reproducibility between runs. Generalized UniFrac distance based PCoA analysis of the sequencing of six samples of mouse ileum mucosa in six to seven runs each with different parameters as described in the **Fig 3A and S2E Fig.** UniFrac weight parameter (Alpha) was set to 0.6 for this analysis.
(PDF)

S4 Fig PCoA analysis of the negative controls. Generalized UniFrac distance based PCoA analysis of the sequencing of 10 samples of mouse mesenteric adipose tissue and 2 x 5 negative controls technical replicates. H20 ext: negative control performed by replacing the tissue sample by molecular grade water in lysis/extraction step of the pipeline. H20: negative control performed by replacing the extracted DNA by molecular grade water in the first step of library preparation. UniFrac weight parameter (Alpha) was set to 0.6 for this analysis.
(PDF)

S1 Table BEI Resources mock communities. Bacterial stain list of the genomic DNA mixture with concentration (16S rDNA copies/μl) of the even and staggered Mixture.
(PDF)

S2 Table Optimal multiplexing indexes. 320 indexes designed to allow a proper calibration of the MiSeq camera and accurate demultiplexing of samples.
(PDF)

## Abbreviations

BEI: biodefense and emerging infections
CPU: central processing unit
DNA: deoxyribonucleic acid
dNTP: deoxyribonucleotide triphosphate
EDTA: ethylene diamine tetra acetic
FLASH: fast length adjustment of short reads
HPLC: high performance liquid chromatography
INRA: institut national de la recherche agronomique
INSERM: institut national de la santé et de la recherche médicale
LB: lysogeny broth
LED: light emitting diode
MAT: mesenteric adipose tissue
NIAID: national institute of allergy and infectious diseases
NIH: national institute of health
NGS: next generation sequencing
OTU: operational taxonomic unit
PCoA: principal coordinates analysis
PCR: polymerase chain reaction
QC: quality control
RAM: random access memory
rDNA: ribosomal deoxyribonucleic acid
RDP: ribosomal database projet
RFLP: restriction fragment length polymorphism
RNA: ribonucleic acid
SEM: standard error of the mean
SOP: standard operating procedure
TBE: tris borate ethylene diamine tetra acetic
UV: ultraviolet
