# Supplementary figures and images for: The Characterization of Novel Tissue Microbiota Using an Optimized 16S Metagenomic Sequencing Pipeline
Source: PLoS One. 2015 Nov 6;10(11):e0142334. doi: 10.1371/journal.pone.0142334 (PMC4636327; doi:10.1371/journal.pone.0142334)

# Supplemental Figure 1

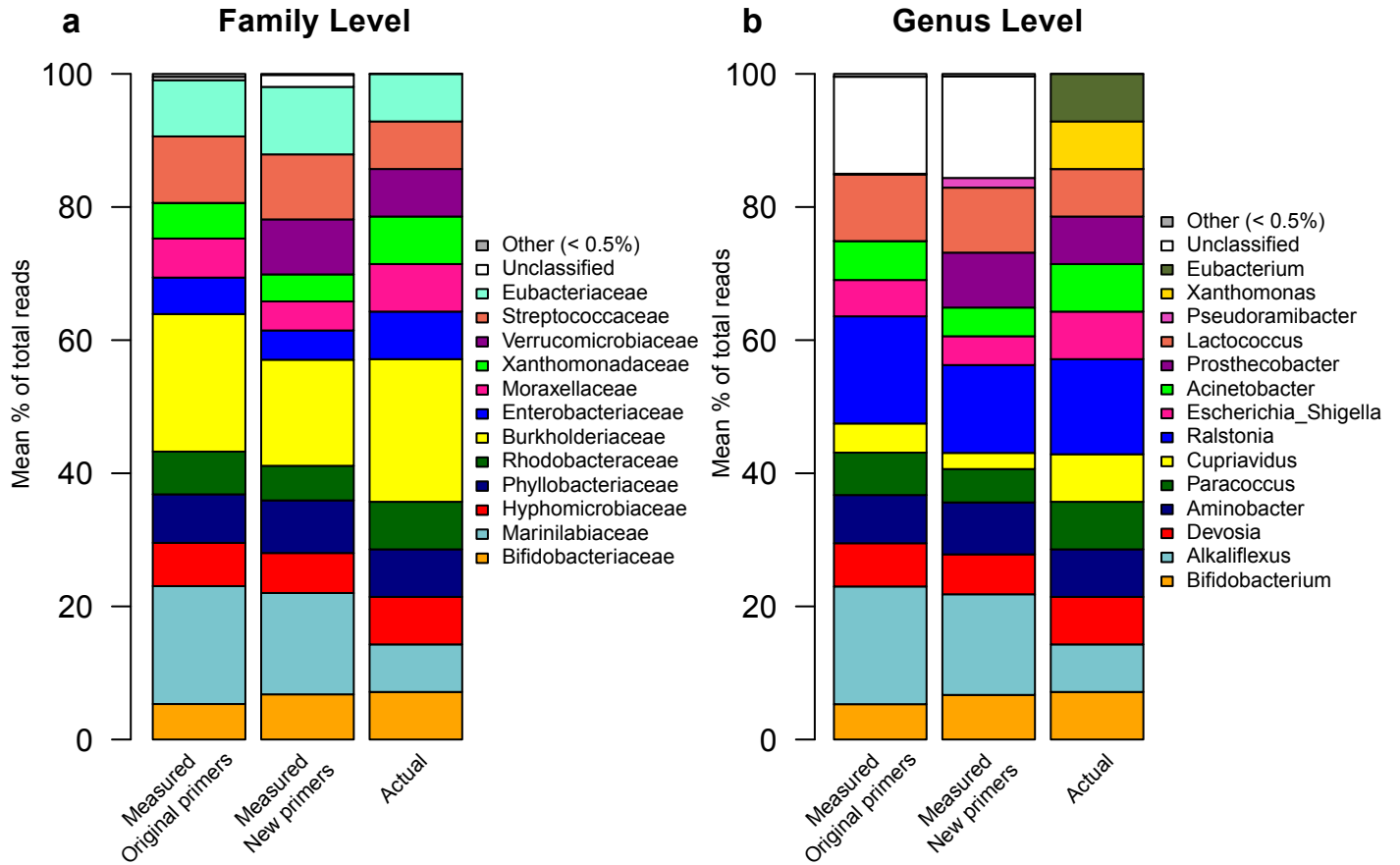

Supplement: S1 Fig — (a) Stacked bar charts showing the actual relative abundance of bacterial families in the plasmid based mock community and the measured relative abundance of the families obtained with the MiSeq sequencing pipeline using either the original primers (described in the methods), or the new primers (designed to amplify also the Verrucomicrobia phylum). (b) The relative abundance as in a, but at the genus taxonomic level. The sequencing was performed in triplicate for all the samples (starting from the extracted DNA); the means of the triplicates are shown on the stacked bar charts. (PDF) [file pone.0142334.s001.pdf]

Supplemental Figure 2

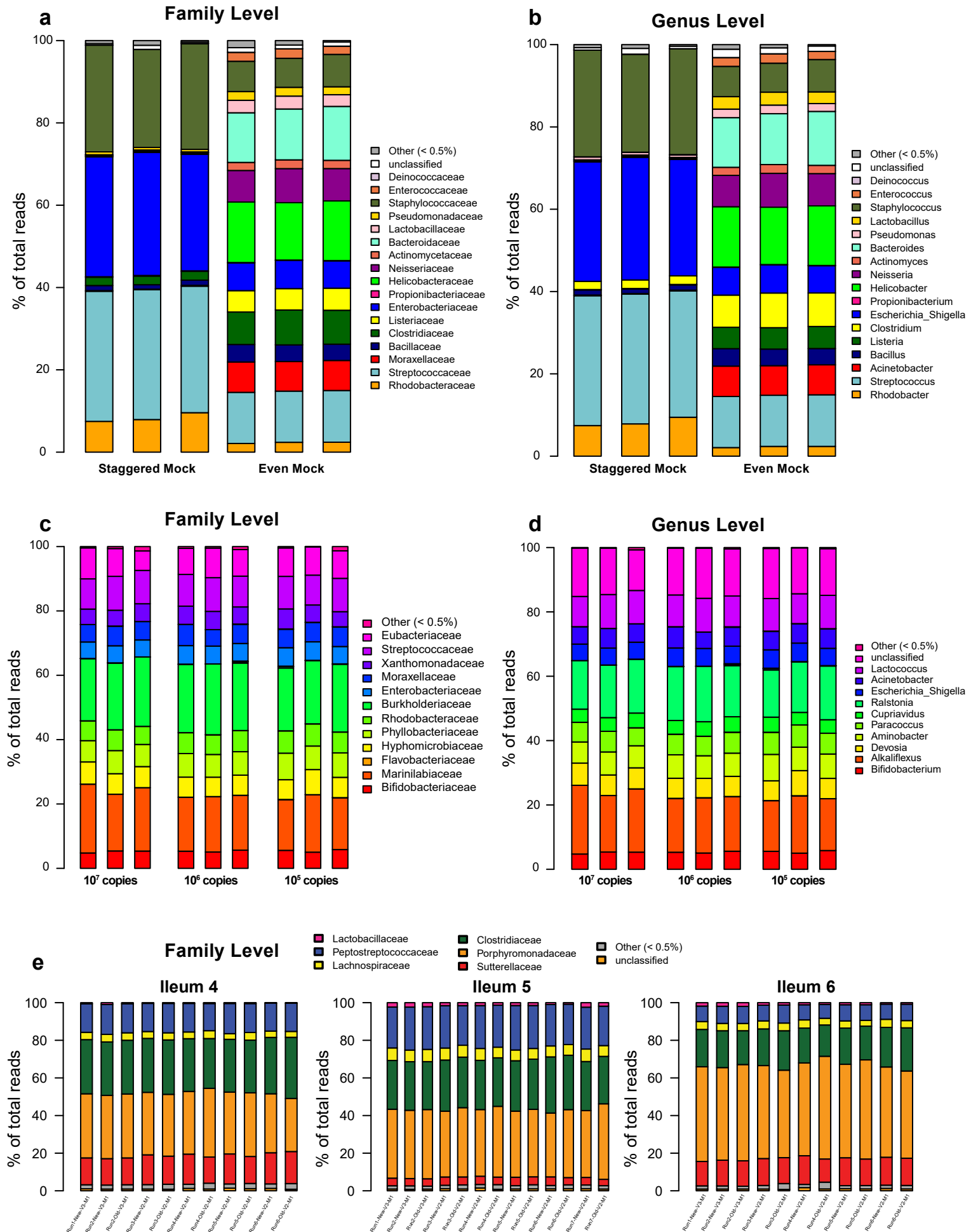

Supplement: S2 Fig — (a-d) Stacked bar charts showing in triplicates the actual relative abundance and the measured relative abundance of the families (a, c) and genus (b, d) of the BEI mock communities (a, b) and our own designed mock communities (c, d). (e) Stacked bar charts showing the relative abundance of bacterial families obtained by sequencing of three samples of mouse ileum mucosa in six to seven runs each with different parameters described in the legend at the bottom left: different runs, new libraries from same extracted DNA or the same libraries already prepared, different MiSeq kit generations and different sequencers. Two experimenters performed the different runs and reagent batch numbers (including Taq polymerase) varied from run to run. (PDF) [file pone.0142334.s002.pdf]

Supplemental Figure 3

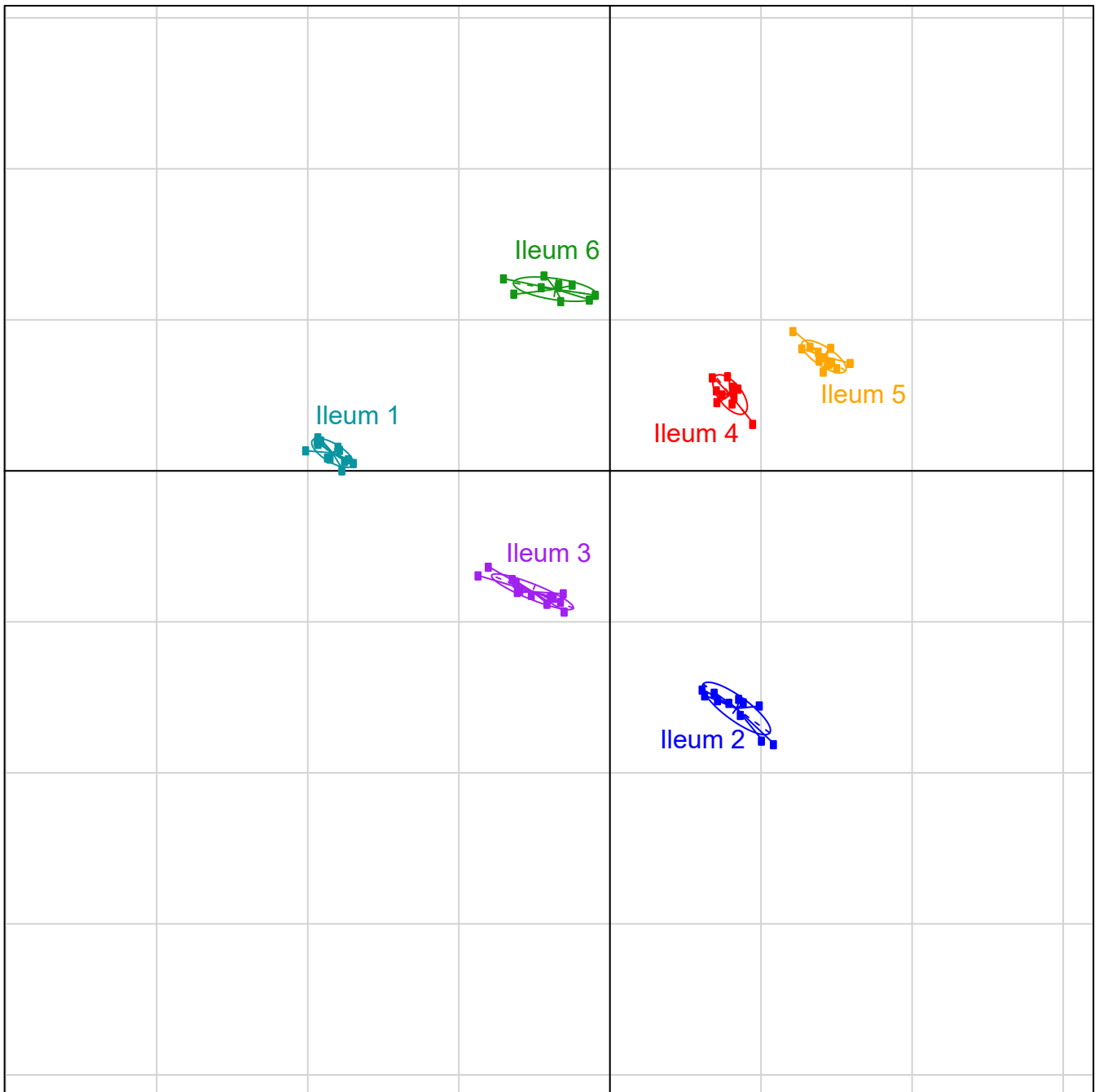

Supplement: S3 Fig — Generalized UniFrac distance based PCoA analysis of the sequencing of six samples of mouse ileum mucosa in six to seven runs each with different parameters as described in the Fig 3A and S2E Fig. UniFrac weight parameter (Alpha) was set to 0.6 for this analysis. (PDF) [file pone.0142334.s003.pdf]

**Supplemental Figure 4**

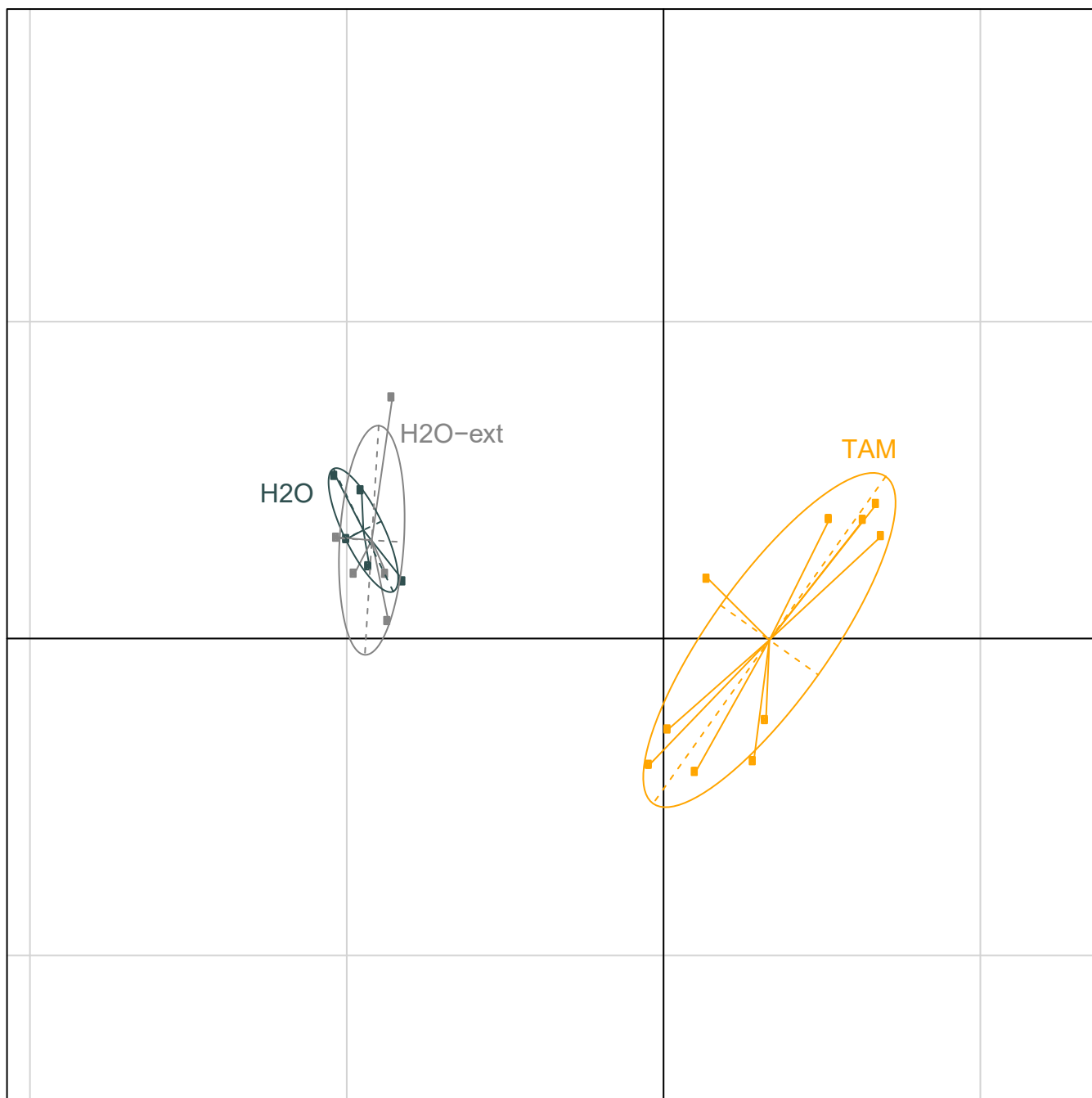

Supplement: S4 Fig — Generalized UniFrac distance based PCoA analysis of the sequencing of 10 samples of mouse mesenteric adipose tissue and 2 x 5 negative controls technical replicates. H20 ext: negative control performed by replacing the tissue sample by molecular grade water in lysis/extraction step of the pipeline. H20: negative control performed by replacing the extracted DNA by molecular grade water in the first step of library preparation. UniFrac weight parameter (Alpha) was set to 0.6 for this analysis. (PDF) [file pone.0142334.s004.pdf]
