# Supplementary material for: The Characterization of Novel Tissue Microbiota Using an Optimized 16S Metagenomic Sequencing Pipeline
Source: PLoS One. 2015 Nov 6;10(11):e0142334. doi: 10.1371/journal.pone.0142334 (PMC4636327; doi:10.1371/journal.pone.0142334)
Supplement: S1 Table — Bacterial stain list of the genomic DNA mixture with concentration (16S rDNA copies/μl) of the even and staggered Mixture. (PDF) [file pone.0142334.s005.pdf]

Supplemental Table 1: BEI Resources mock communities

Species	Family	Phylum	Copies o f16s rDNA/µl	
			Even Mock	Staggered Mixture
Acinetobacter baumannii	Moraxellaceae	Proteobacteria (Gamma)	10^5	10^4
Actinomyces odontolyticus	Actinomycetaceae	Actinobacteria	10^5	10^3
Bacillus cereus	Bacillaceae	Firmicutes	10^5	10^5
Bacteroides vulgatus	Bacteroidaceae	Bacteroidetes/Chlorobi	10^5	10^3
Clostridium beijerinckii	Clostridiaceae	Firmicutes	10^5	10^5
Deinococcus radiodurans	Deinococcaceae	Deinococcus-Thermus	10^5	10^3
Enterococcus faecalis	Enterococcaceae	Firmicutes	10^5	10^3
Escherichia coli	Enterobacteriaceae	Proteobacteria (Gamma)	10^5	10^6
Helicobacter pylori	Helicobacteraceae	Proteobacteria (Epsilon)	10^5	10^4
Lactobacillus gasseri	Lactobacillaceae	Firmicutes	10^5	10^4
Listeria monocytogenes	Listeriaceae	Firmicutes	10^5	10^4
Neisseria meningitidis	Neisseriaceae	Proteobacteria (Beta)	10^5	10^4
Propionibacterium acnes	Propionibacteriaceae	Actinobacteria	10^5	10^4
Pseudomonas aeruginosa	Pseudomonadaceae	Proteobacteria (Gamma)	10^5	10^5
Rhodobacter sphaeroides	Rhodobacteraceae	Proteobacteria (alpha)	10^5	10^5
Staphylococcus aureus	Staphylococcaceae	Firmicutes	10^5	10^6
Staphylococcus epidermidis	Staphylococcaceae	Firmicutes	10^5	10^5
Streptococcus agalactiae	Streptococcaceae	Firmicutes	10^5	10^6
Streptococcus mutans	Streptococcaceae	Firmicutes	10^5	10^3
Streptococcus pneumoniae	Streptococcaceae	Firmicutes	10^5	10^5
