# Supplementary material for: The Characterization of Novel Tissue Microbiota Using an Optimized 16S Metagenomic Sequencing Pipeline
Source: PLoS One. 2015 Nov 6;10(11):e0142334. doi: 10.1371/journal.pone.0142334 (PMC4636327; doi:10.1371/journal.pone.0142334)
Supplement: S2 Table — 320 indexes designed to allow a proper calibration of the MiSeq camera and accurate demultiplexing of samples. (PDF) [file pone.0142334.s006.pdf]

Supplemental Table 2: Optimal multiplexing indexes

AAACAA	ATAAGA	CGCGTG	GCCGGT	TATCAT
AAACTC	ATACTG	CGCTCT	GCCTAA	TATCCC
AAAGCG	ATATAA	CGGAAC	GCGATG	TATCGA
AAAGTA	ATATCC	CGGCAT	GCGCCC	TATGCG
AAATGT	ATCGGG	CGGGAG	GCTATC	TATTGG
AAATTG	ATCGTT	CGTCGC	GCTCAA	TCACAC
AACCAT	ATCTAT	CGTGCG	GCTTCG	TCAGCG
AACCGA	ATGAAC	CGTTAA	GGAAGA	TCATCA
AACGCA	ATGCTT	CGTTGG	GGACGG	TCATGT
AACGTC	ATGTCA	CGTTTC	GGACTT	TCCAGA
AACTAG	ATTACA	CTACAT	GGAGAG	TCCATT
AACTTA	ATTAGG	CTACCG	GGAGGT	TCCCCA
AAGCTA	ATTCTC	CTACGC	GGATCA	TCCTTA
AAGGCC	ATTGAG	CTACTA	GGATTC	TCGAAC
AAGGTG	ATTTGT	CTAGAG	GGCAAT	TCGAGG
AAGTAA	CAACAG	CTAGGA	GGCCAA	TCGCGC
AAGTGG	CAACGA	CTAGTC	GGCCTC	TCGGCC
AATAAT	CAATGC	CTATGG	GGCGTA	TCGTTC
AATATG	CAATTA	CTCATA	GGCTGC	TCTATG
AATCAC	CACACT	CTCCAG	GGCTTG	TCTCGG
AATCCG	CACCCA	CTCCCC	GGGGGA	TCTCTC
AATGAA	CACCGG	CTCGGT	GGGGTG	TCTGAT
AATGGT	CACCTC	CTCTAC	GGGTCT	TCTTAA
AATTCT	CACTAA	CTCTCG	GGTAGC	TCTTCT
AATTGC	CACTGT	CTGAGG	GGTATA	TGAATA
ACAAAA	CAGAAA	CTGCAC	GGTCAC	TGACCT
ACACCG	CAGAGC	CTGGTT	GGTCCG	TGAGCA
ACACGC	CAGATG	CTGTAA	GGTGTT	TGATGC
ACAGAC	CAGCGT	CTGTGC	GGTTAT	TGATTT
ACAGTT	CAGGAC	CTTAAA	GTAACA	TGCACG
ACATAT	CAGGCG	CTTGAC	GTAGAA	TGCAGT
ACATTA	CAGTCT	CTTGCA	GTAGTT	TGCCAG
ACCAGG	CAGTGA	CTTGGG	GTCAAA	TGCCTT
ACCCCC	CAGTTC	GAACAC	GTCAGG	TGCGCT
ACCGTG	CATCCT	GACAGT	GTCATT	TGCGGG
ACCTCA	CATGAG	GACATC	GTCCTA	TGCTGA
ACGAAT	CATGTT	GACGAT	GTCGGC	TGGATT
ACGAGA	CCAACA	GACTAC	GTCGTG	TGGGTA
ACGATC	CCAAGC	GACTCG	GTGAAT	TGGTAG
ACGGTA	CCAGCC	GACTTT	GTGACG	TGGTCA
ACTACT	CCATTG	GAGAAC	GTGCCA	TGGTGT
ACTCGA	CCCAAA	GAGAGG	GTGCTG	TGTAAA
ACTGCG	CCCACG	GAGATA	GTGGGG	TGTCGT
ACTTTT	CCCCAT	GAGCAA	GTGTAG	TGTCTA
AGAAGT	CCCGAG	GAGCTT	GTGTTT	TGTGAG
AGAATC	CCCGGC	GAGGGC	GTTACC	TGTGCC
AGAGGG	CCCGTT	GAGTTG	GTTAGT	TGTTCG
AGATAG	CCCTCC	GATAGA	GTTCGC	TTAAAT
AGCCCT	CCCTGG	GATCTG	GTTCTT	TTAAGG
AGCCTG	CCGACC	GATGAC	GTTGCG	TTACTC
AGCGAC	CCGGGT	GATGCT	GTTTCT	TTAGCT
AGCGGT	CCGTAG	GATTCA	TAAATG	TTCAAC
AGGCGC	CCGTCA	GCAAAT	TAACTT	TTCCGT
AGGGAT	CCTCCC	GCAATA	TAAGAC	TTCGAG
AGGGCA	CCTCTT	GCACGT	TAAGGG	TTCGCA
AGGTTC	CCTGCT	GCACTG	TACAAG	TTCTTG
AGTAAG	CCTTGA	GCAGCT	TACCAA	TTGAAG
AGTATT	CGAAGG	GCATAC	TACCTG	TTGACT
AGTCGG	CGAGAA	GCATGG	TACGGC	TTGCCC
AGTGCT	CGATAC	GCCAAG	TACTCA	TTGCTA
AGTGTC	CGCACA	GCCACA	TAGATC	TTGTGA
AGTTGA	CGCATT	GCCCAC	TAGGAA	TTTCCG
AGTTTG	CGCCAC	GCCCGA	TAGGCT	TTTGTA
ATAACG	CGCGCC	GCCGCG	TAGTAC	TTTTTC
